# Regulatory T cells and IgE expression in duodenal mucosa of *Strongyloides stercoralis* and human T lymphotropic virus type 1 co-infected patients

**DOI:** 10.1371/journal.pntd.0007415

**Published:** 2019-06-06

**Authors:** Luis Malpica, A. Clinton White, Cristina Leguia, Natalia Freundt, Nicolas Barros, Cesar Chian, E. Antonio Antunez, Martin Montes

**Affiliations:** 1 Instituto de Medicina Tropical ‘Alexander von Humboldt’, Facultad de Medicina “Alberto Hurtado”, Universidad Peruana Cayetano Heredia, Lima, Peru; 2 Department of Internal Medicine, Division of Infectious Diseases, University of Texas Medical Branch at Galveston, Texas, United States of America; 3 Departamento de Patología, Hospital Nacional Arzobispo Loayza in Lima, Peru; PUCRS, BRAZIL

## Abstract

**Background:**

*Strongyloides stercoralis* is an intestinal nematode unique in its ability to replicate in the human host, allowing ongoing cycles of autoinfection, persisting for decades within the same host. Although usually asymptomatic, overwhelming infections can occur in *Strongyloides* and HTLV-1 co-infected individuals (SS/HTLV-1). Regulatory T cells (Tregs) are able to blunt specific Th2 responses necessary to control the parasite. We previously reported that peripheral blood Tregs are increased in SS/HTLV-1 and correlate with low Th2 responses. We hypothesized that Tregs are also increased at the site of infection in duodenal mucosa.

**Methods:**

Paraffin embedded duodenal biopsies were obtained from 10 SS/HTLV-1 patients, 3 controls with non-parasitic chronic duodenitis, and 2 healthy controls. Immunohistochemistry was performed using monoclonal antibodies against human CD3, CD8, IgE and FoxP3. The number of cells were counted using a conventional light microscope. The number of CD3+, CD8+, FoxP3+ and IgE positive cells per 0.35 mm^2^ was measured using ImagePro Plus software comparing areas adjacent or distant from parasite material.

**Results:**

In patients with SS/HTLV-1, T lymphocyte counts and CD8+ cells were lower in areas adjacent to the parasite compared to non-adjacent areas (CD3+: adjacent: 6.5 [Interquartile range (IQR: 2.8–12.3)]; non-adjacent: 24.5 [IQR: 20.9–34.4]; Mann-Whitney p = 0.0003; CD8+: adjacent: 4.5 [IQR: 2.3–11.8]; non-adjacent: 21 [IQR: 15.3–42.9]; Mann-Whitney p = 0.0011). Tregs cells in the intestines (FoxP3+ expressing cells) were increased in patients with SS/HTLV-1 compared with patients with chronic duodenitis (SS/HTLV-1: 1.5 [IQR: 0.7–2.3]; duodenitis controls: 0 [range 0–0.7]; healthy controls: 0; Mann-Whitney p = 0.034). There was also a trend towards fewer eosinophils adjacent to the parasites. Among SS/HTLV-1 patients the number of IgE expressing cells was increased for in areas not adjacent to the parasite compared to non-adjacent areas (ANOVA, p = 0.001).

**Conclusions:**

Our data shows increased Treg cell numbers localized adjacent to the parasites in the duodenum SS/HTLV-1 patients. In addition, other T lymphocytes and IgE expressing cells were decreased adjacent to the parasites, suggesting an important role for Tregs in down-regulating local parasite effector responses.

## Author summary

Strongyloidiasis is a parasitic infection found worldwide in warm, moist climates. In most people, *Strongyloides* causes a mild and chronic infection with few symptoms. However, some patients, including those infected with the Human T Lymphotrophic Virus 1 (HTLV-1), can get uncontrolled disease called hyperinfection, which can be fatal. To help determine why this infection is so serious in co-infected patients, we studied biopsies obtained from the small intestine from patients with both infections and compared them to control biopsies. The biopsies from patients with both *Strongyloides* and HTLV-1 had increased numbers of white blood cells in their biopsies. In particular they displayed a type of lymphocyte that downregulates immune responses. Some of them had increased numbers of cells called eosinophils in the intestines. These cells can help eliminate *Strongyloides*. However, they were not found near the parasite, suggesting that something near the parasite was suppressing the host response that would control infection. These studies provide more evidence that HTLV-1 suppresses the host response that controls *Strongyloides* and that the suppression occurs at the site of infection in the small intestines.

## Introduction

*Strongyloides stercoralis* (Strongyloides), is a soil-transmitted nematode endemic in most moist tropical and subtropical areas worldwide [1–5]. It is highly endemic in tropical Asia, sub-Saharan Africa, and most of Latin America with some estimates of over 300 million people infected worldwide [6]. Much of the population in parts of southeast Asia and the Amazon is infected [6][7]. *Strongyloides* has a unique life cycle, which facilitates autoinfection and chronic infection. Female parasites infect the duodenal mucosa, where they deposit eggs. The eggs hatch in the mucosa and release larvae into the intestinal lumen. A portion of the larvae mature into infectious larvae prior to exiting the host allowing reinvasion through the intestines or perianal region. This leads to cycles of autoinfection, which can persist for decades even after subjects have left the endemic areas. Most infections cause few or no symptoms and are controlled by the host [5]. However, in a subset of patients, the autoinfection cycle can accelerate leading to hyperinfection. which can present with pulmonary and/or gastrointestinal hemorrhage, bacterial superinfection, and death. The main risk factors for hyperinfection include treatment with corticosteroids or co-infection with the retrovirus Human T cell Lymphotropic Virus 1 (HTLV-1) [1]. Both HTLV-1 and *Strongyloides* infections are endemic in Peru so that co-infection is also common [7, 8].

While the mechanisms are incompletely understood, eosinophils and neutrophils appear to be critical for prevention of hyperinfection [9, 10]. Chronic infection is associated with a Th2 type of immune response [11]. The expression of Th2 cytokines, especially IL-4 and IL-5, drives the activation, expansion and migration of eosinophils and production of specific antibody [12]. Eosinophils both directly kill the parasites and also facilitate the production of specific antibody, which enhances neutrophil-mediated parasite killing [9].

HTLV-1 is a retrovirus, which infects CD4+ T cells and profoundly affects host responses. HTLV-1 immortalizes cells leading, in turn, to spontaneous cytokine production [8]. This cytokine response including Interferon gamma, transforming growth factor β, and Interleukin 10, each of which suppresses eosinophil numbers. Regulatory T cells (Tregs) are a CD4+ T cell subset characterized by their constitutive expression of FoxP3. Tregs down-regulate both innate and adaptive immune responses. This function is important in prevention of over-exuberant responses and overwhelming fibrosis, seen with an unbalanced Th2 response. Our group demonstrated that patients with HTLV-1 have increased numbers of circulating Tregs. *Strongyloides* co-infection further increased levels of circulating Tregs compared to patients with either HTLV-1 or *Strongyloides* alone [13]. The increased number of Tregs is associated with a blunted antigen-driven IL-5 expression, and significantly lower peripheral eosinophil counts in HTLV-1 co-infected patients compared with patients with only *Strongyloides* infection [13]. On the other hand, *Strongyloides* leads to increased HTVL-1 proviral load and may stimulate oligoclonal expansion of infected cells [14]. Thus, co-infection promotes a vicious cycle in which HTLV-1 leads to an increased parasite burden of *Strongyloides* and *Strongyloides* accelerates HTLV-1 proliferation. However, there are no data on the host response to *Strongyloides* at the site of infection in the intestines. The description of the local response would add significant information to the understanding of the immune responses to *Strongyloides*. In the present study we studied Tregs, eosinophils and IgE in the duodenal mucosa of *Strongyloides* and HTLV-1 co-infected individuals, compared to controls.

## Methods

### Study participants

We studied formalin-fixed, paraffin-embedded duodenal tissues from ten patients with a clinical history and pathological findings of duodenitis caused by *Strongyloides* infection. The duodenal biopsies were obtained from the archives of the pathology department of Hospital Nacional Arzobispo Loayza and Hospital Nacional Cayetano Heredia, Lima, Peru. All *Strongyloides* patients were seropositive for HTLV-1 infection. In addition, we obtained duodenal biopsies from 3 patients with a histopathologic diagnosis of chronic non-parasitic duodenitis. Biopsies revealed duodenal inflammation without an obvious cause. We limited controls to those with both stool studies and biopsies without demonstrable parasites. We also included two duodenal biopsies of healthy individuals, both with no medical history of immunosupressive condition, parasitic infection or recent travel to endemic areas. In both, HIV and HTLV-1/2 serologies were negative. Demographic and clinical information were obtained when available. We were unable to identify any biopsies from patients with just *Strongyloides* (who are usually identified without requiring endoscopy or biopsy) or only HTLV-1 (who rarely present with symptoms requiring endoscopy).

### Ethics

The Institutional Review Board (Comité Institucional de Etica) of the Universidad Peruana Cayetano Heredia in Lima, Perú, approved the study protocol (SIDISI code 55015–2010). All subjects were adults (defined in Peru as >18 years old). Most specimens were archived specimens from patients who had undergone endoscopy and biopsy for clinical indications. Extra tissues were used and records were reviewed following the approved protocol. Normal volunteers underwent endoscopy and had provided written consent prior to the procedures.

### Histopathologic study

Using a hemotoxylin-eosin (H&E) stain, we evaluated the degree of inflammatory infiltrate, the proportion of predominant inflammatory cells according to their location in the mucosa and the degree of edema and villous atrophy. Additionally, the number of *Strongyloides* larvae present per 100x field were countred for the entire slide (parasite burden), as well as the presence of eosinophilic infiltration and ulceration.

### Immunohistochemical assays

Four micron sections of the tissues were mounted on glass slides (Fisher Scientific, Pittsburgh, PA). The sections were deparaffinized and rehydrated in graded alcohol, washed and placed in water. Endogenous peroxidase activity was blocked by covering the slides with 3% hydrogen peroxidase for 10 minutes. Afterwards, the slides were washed with phosphate-buffered saline (PBS) 0.01 M pH 7.4. After heat mediated antigen retrieval (with Tris buffer 0.05 M pH 9.0 at 95 °C for 40 minutes), sections were washed thrice with PBS for 5 minutes. Primary antibody incubation was performed in 1 hour using the following monoclonal antibodies: 1:100 dilution of mouse anti-human CD3 (Dako, F7.2.38, 260 mg/L, Agilent Dako, Santa Clara, CA); 1:100 dilution of mouse anti-human CD8 (Dako, C8/144B, 200 mg/L, Agilent-Dako), 1:100 dilution of anti-human IgE (Goat polyclonal secondary antibody to Human IgE—epsilon chain Abcam, Cambridge, UK, ab9159, 1 mg/ml) and 1:10 mouse anti-human FoxP3 (anti-Human FoxP3 Biotin eBioscience, San Diego, CA, PCH101, 0.5 mg/mL). Sections were washed with PBS buffer for 5 minutes. The slides were then incubated with secondary antibody, biotinylated anti-mouse IgG (Vector Laboratories, Cambridgeshire, UK) for 20 min followed by streptavidin peroxidase reagent for 30 min. After washing the slides in Tris Buffer 0.1 M pH 7.6 solution (5 minutes, 2 washings), sections were incubated with 3,3’-diaminobenzidine (DAB) for 2 to 5 minutes. Sections were then washed with running tap water and counterstained with haematoxylin, dehydrated and mounted using cover slips and Canada Balsam.

### Cell quantification

The numbers of eosinophils, IgE+, CD3+, CD8 + and FoxP3 + cells were counted using a conventional light microscope at a magnification of 400X. At least 500 cells were counted in each biopsy specimen. Images were taken and the area was measured using the ImagePro Plus software (Media Cybernetics, Rockville, MD). Positive cells in the lamina propria were counted as the percentage of DAB-stained cells compared to the total number of nucleated cells in the lamina propria. Positive cells were counted and the data expressed as the number of cells/0.35mm^2^. The relative numbers of cells expressing IgE, CD3, CD8, or FoxP3 in the duodenal epithelium were quantified by counting the number of positively stained cells in a given length of epithelium. At least 200 positively stained lymphocytes were counted in each biopsy specimen. Separate calculations were made for the villi and crypts, which were identified based on microscopy. Areas within a 0.335 mm radius of the parasite material on the slides were classified as adjacent to the parasite. The distribution of IgE+ expressing cells in the lamina propria of the villi and in the lamina propria of the crypt was compared in areas adjacent and non-adjacent to the parasite. For the 3 patients with eosinophilic infiltration, eosinophils were counted from slides stained with hematoxylin and eosin comparing 100x fields that did or did not have visible parasites.

### Analysis

Our central hypothesis was that Tregs would be localized to the duodenum in co-infected patients. We further hypothesized that areas with increased Tregs would have lower numbers of eosinophils. The latter included planned separate analyses of crypts and villi. The relative numbers of eosinophils and cells expressing IgE, CD3, CD8, or FoxP3 in the duodenal epithelium are presented as medians with interquartile ranges. We used Mann-Whitney test and ANOVA to assess differences between *SS*/HTLV-1 patients, chronic duodenitis patients and healthy individuals. After initial observations suggested sparing of inflammatory cells adjacent to the parasites, we used Mann-Whitney test and ANOVA to compare areas adjacent or non-adjacent to the parasites. P values <0,05 were considered statistically significant. Analysis were performed using SPSS 19.0 (IBM SPSS statistical analysis software, CA, USA).

## Results

### Clinical features

We identified duodenal biopsies from 10 patients that demonstrated *Strongyloides* parasites. Six were from men and 4 from women. The average age was 33 years (range 16–61). The main clinical manifestations included weight loss, abdominal pain, fatigue, gastrointestinal bleeding, diarrhea, anemia, and small bowel obstruction. All *Strongyloides* patients were positive for HTLV-1 antibody. Seven were HIV negative. The other 3 were not tested for HIV, but had no clinical evidence of AIDS-related conditions in their medical records. Stool samples for ova and parasites identified *Strongyloides* rhabditiform larvae in 1 case; the other 9 had negative stool studies, Indeed, the reason for endoscopy and biopsy was in part to look for causes of symptoms, in the absents of a diagnosis from stool studies. Of the 8 with blood counts available, 5 had eosinophilia. The median eosinophil count was 647/mm^3^, only 2 had eosinophil counts above 1,000/mm^3^. All of the control patients had a normal laboratory profile and had ≥3 stool samples negative for parasites. All duodenitis controls were seronegative for both HTLV-1/2 and HIV antibodies. Clinical and demographic data are outlined in Table 1.

**Table 1:** Clinical characteristics of patients with HTLV-1/*Strongyloides* co-infection and chronic duodenitis controls.

	Age/Sex	Clinical Presentation	Retroviral serology	Stool Ova/ parasites	WBCs/mm^3^	Lymphocytes/mm^3^	Eosinophils/mm^3^
1	47/M	Weight loss, fatigue	HTLV-1 (+), HIV (-)	Negative	12,600	2,646	1,764
2	20/F	Weight loss, fatigue	HTLV-1 (+), HIV (-)	Negative	10,490	1,259	1,154
3	61/F	Weight loss, fatigue, edema	HTLV-1 (+), HIV (-)	Negative	10,500	2,310	630
4	23/M	Weight loss, abdominal pain, diarrhea	HTLV-1 (+), HIV (-)	Negative	9,190	2,757	643
5	36/M	Weight loss, abdominal pain	HTLV-1 (+)	Negative	6,000	1,740	840
6	33/M	Weight loss, abdominal pain	HTLV-1 (+)	Negative			
7	18/M	Small bowel obstruction, abdominal pain	HTLV-1 (+), HIV (-)	Larvae			
8	55/F	Weight loss, anemia	HTLV-1 (+)	Negative	3900	936	0
9	16/F	Abdominal pain, GI bleeding	HTLV-1 (+), HIV (-)	Negative	7420	913	37
10	25/M	Abdominal pain, GI bleeding	HTLV-1 (+), HIV (-)	Negative	5070	1222	106
**Chronic duodenitis controls**							
11	26/M	Abdominal pain	HTLV-1 (-), HIV (-)	Negative	5850	1697	59
12	56/F	Chronic diarrhea, dyspepsia	HTLV-1 (-), HIV (-)	Negative	4800	960	0
13	78/M	Chronic diarrhea	HTLV-1 (-), HIV (-)	Negative	7670	844	77
**Normal controls**							
14	56/F	None					
15	19/M	None					

### Endoscopic and histopathological findings

Gross endoscopic abnormalities were observed in 9/10 SS/HTLV-1 patients. Abnormalities included erythematous mucosa (5), edematous mucosa (4), hemorrhage (2), stenosis (2), ulceration (2) and pale villi (1). Most patients had more than one finding. In the chronic duodenitis controls, 2 had a normal upper endoscopy. The other one had mild mucosal erythema. The histopathological findings in SS/HTLV-1 patients and controls are summarized in Table 2. Villous atrophy was identified in all cases with *Strongyloides co*-infection with severe villous atrophy in 8, moderate atrophy in 1 and mild atrophy in 1. Villous atrophy was also identified in the controls. Most of the cases with *Strongyloides co*-infection had mild to moderate mucosal edema. All controls with chronic duodenitis had moderate mucosal edema. Three (30%) of the *Strongyloides*/HTLV-1 co-infection patients had severe eosinophilic infiltration. None of the controls had significant eosinophil infiltration.

**Table 2:** Histopathological findings in duodenal biopsies from S. stercoralis co-infected patients and duodenitis controls.

	Lamina propria infiltration	Predominant inflammatory cells in lamina propria		Villous atrophy	Edema	Eosinophils	Ulceration	Larvae/100x
		Within villi	Below villi					
	**Strongyloides/HTLV-1 Cases**							
1	Moderate	Plasma cells	Lymphocytes	Severe	Mild	Yes	No	Moderate
2	Moderate	Plasma cells	Lymphocytes	Severe	Moderate	Yes	No	Moderate
3	Moderate	Plasma cells	Plasma cells	Moderate	Mild	No	No	Severe
4	Moderate	Plasma cells	Lymphocytes	Severe	Moderate	No	No	Mild
5	Severe	Plasma cells	Lymphocytes	Mild	Moderate	No	No	Mild
6	Moderate	Plasma cells	Lymphocytes	Severe	Moderate	No	No	Mild
7	Moderate	Plasma cells	Lymphocytes	Severe	Severe	No	No	Severe
8	Moderate	Plasma cells	Lymphocytes	Severe	Mild	No	No	Severe
9	Severe	Plasma cells	Lymphocytes	Severe	Mild	Yes	No	Moderate
10	Moderate	Neutrophils	Lymphocytes	Severe	Severe	No	Yes	Moderate
	**Chronic Duodenitis controls**							
11	Severe	Plasma cells	Plasma cells	Mild	Moderate	No	No	None
12	Moderate	Plasma cells	Plasma cells	Severe	Moderate	No	No	None
13	Moderate	Plasma cells	Plasma cells	Moderate	Moderate	No	No	None

Larva burden was graded as low (1-5/100x field), moderate (6-10/100x field), or high (>10/100x field).

The degree of inflammatory cell infiltration in *Strongyloides* co-infection ranged from moderate infiltration in 8 patients to severe infiltration in 2 patients. In the chronic duodenitis controls, 2 had moderate infiltration and 1 severe infiltration. In the *Strongyloides* co-infected individuals, the inflammatory cell population was variable. In the lamina propria within the villi, most patients (9, 90%) had predominantly plasma cells. The other one had ulceration and mainly polymorphonuclear cells. In the lamina propria below the crypts, lymphocytes were the predominant inflammatory cell in 9. All chronic duodenitis controls had predominantly plasma cells throughout the lamina propria.

Most of the cases had 6 to 10 *Strongyloides* larvae observed in each 100x (Table 2). Three (30%) patients had more than 10 larvae observed for each 100x field. These 3 patients had low numbers of eosinophils surrounding the parasite.

### Immunohistochemistry staining

**Fig 1:**
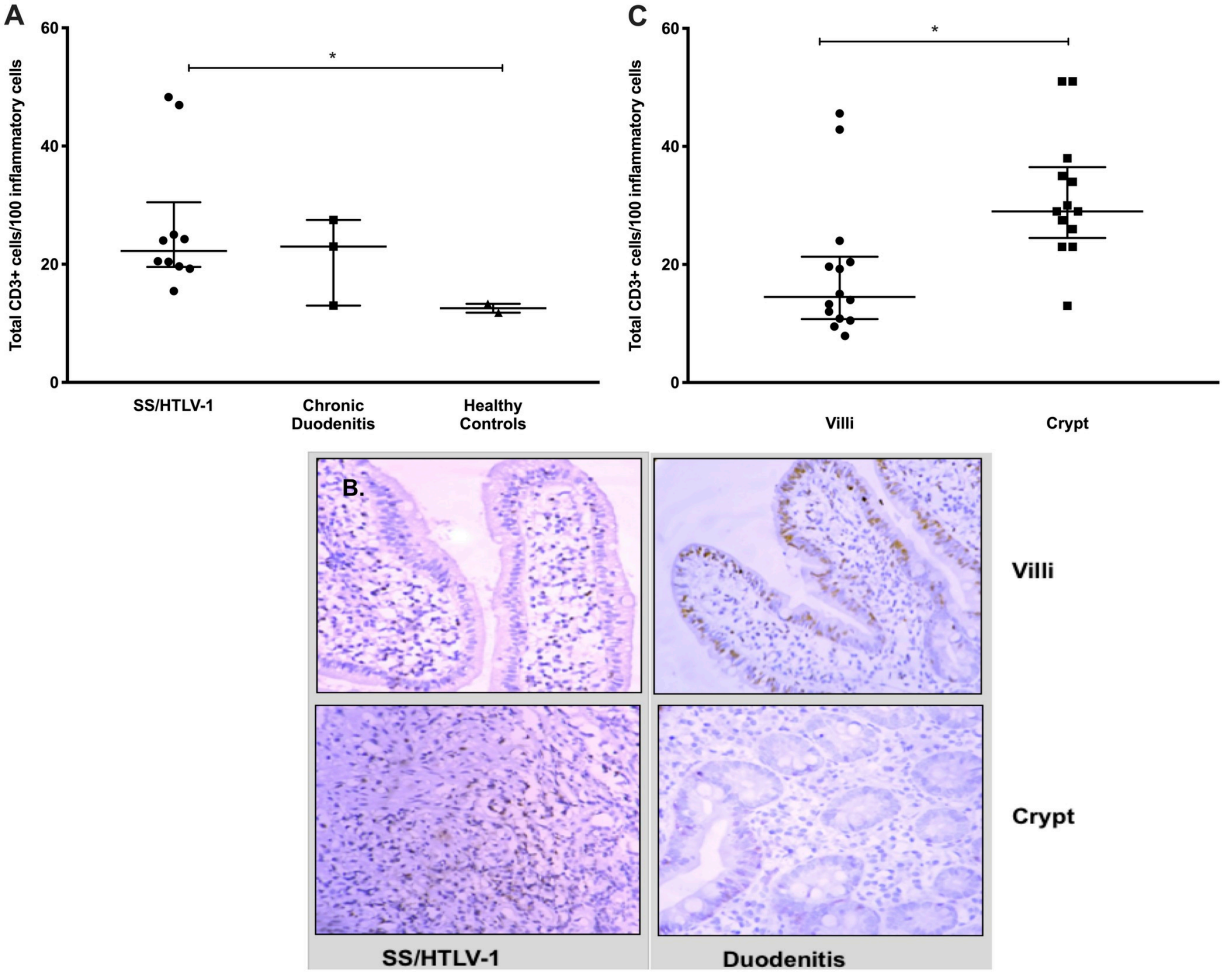
Number and localization of CD3+ T cells in intestinal biopsies from patients with HTLV-1 and Strongyloides co-infection (SS/HTLV-1) compared to controls. Panel A. The number of CD3+ T cells was quantified using ImagePro Plus software demonstrating similar total numbers in SS/HTLV-1 and chronic duodenitis controls, but the numbers were increased compared to healthy controls (*p = 0.03, Mann-Whitney). Panel B. Immunohistochemistry staining for CD3 (positive cells identified by the brown color) showing expression localized to the villi in duodenitis controls and the crypts for SS/HTLV-1. Panel C. In SS/HTLV-1, the CD3+ cells were significantly increased in crypts compared to villi. Long horizontal lines are the medians, vertical line and short horizontal lines demonstrate the interquartile range. (* P<0.004, Mann-Whitney).

**Fig 2:**
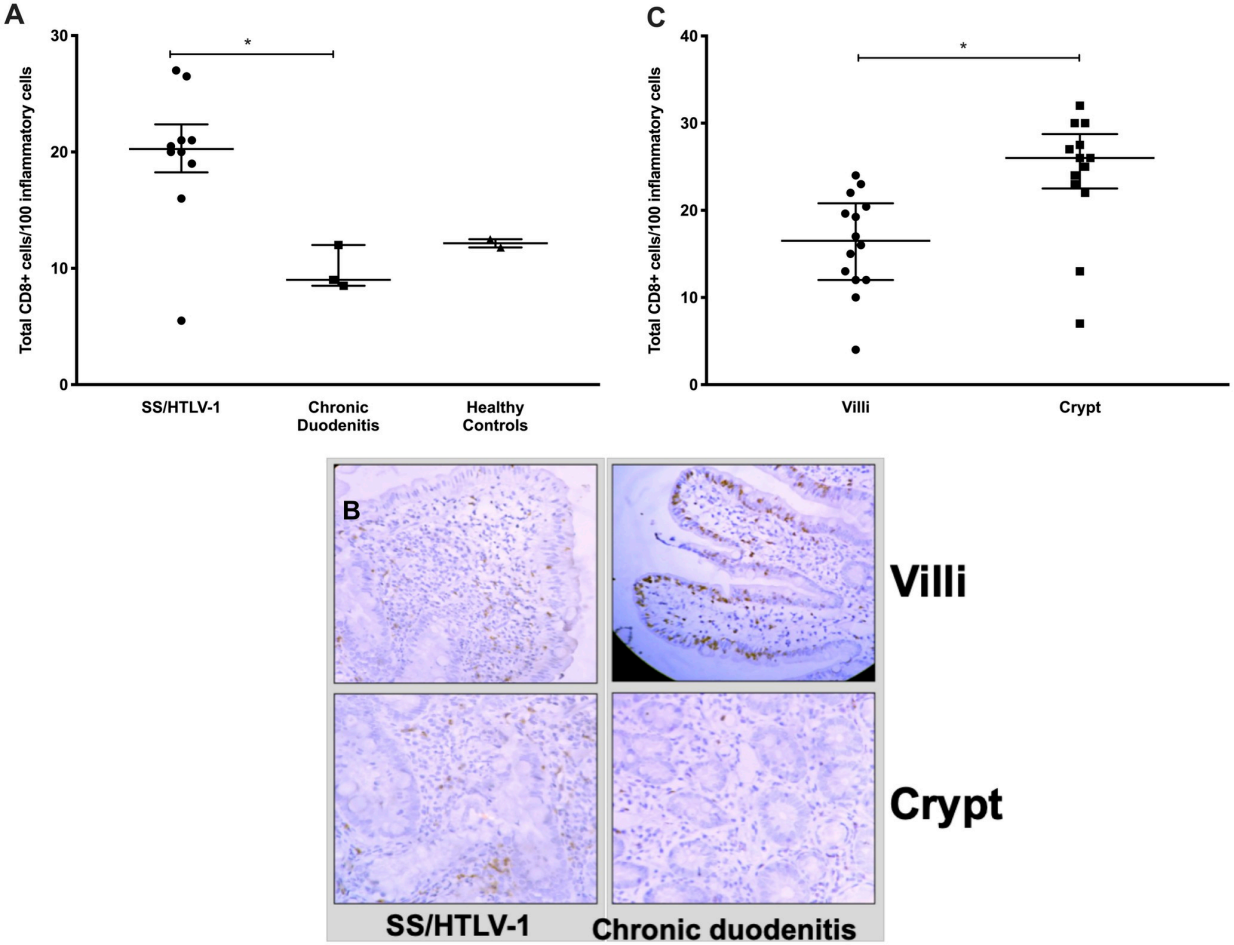
Number and localization of CD8+ cells in intestinal biopsies from patients with HTLV-1 and *Strongyloides* (SS/HTLV-1) compared to controls. Panel A. The number of CD8+ T cells was quantified using ImagePro Plus software demonstrating increased numbers in SS/HTLV-1 compared to chronic duodenitis controls (Mann-Whitney p = 0.04). Panel B. Immunohistochemistry staining for CD8 in biopsies from SS/HTLV-1 patients and chronic duodenitis controls (positive cells identified by the brown color). Distribution in the villi is illustrated on the top panels and between the crypts in the bottom panels. Panel C. Graph demonstrating differential localization of CD8+ cells for SS/HTLV-1 patients with significantly increased expression in the crypts compared to the villi (Mann-Whitney p<0.003). Long horizontal lines are the medians, vertical lines and short horizontal lines demonstrate the interquartile range. * marks statistically significant differences.

**Fig 3:**
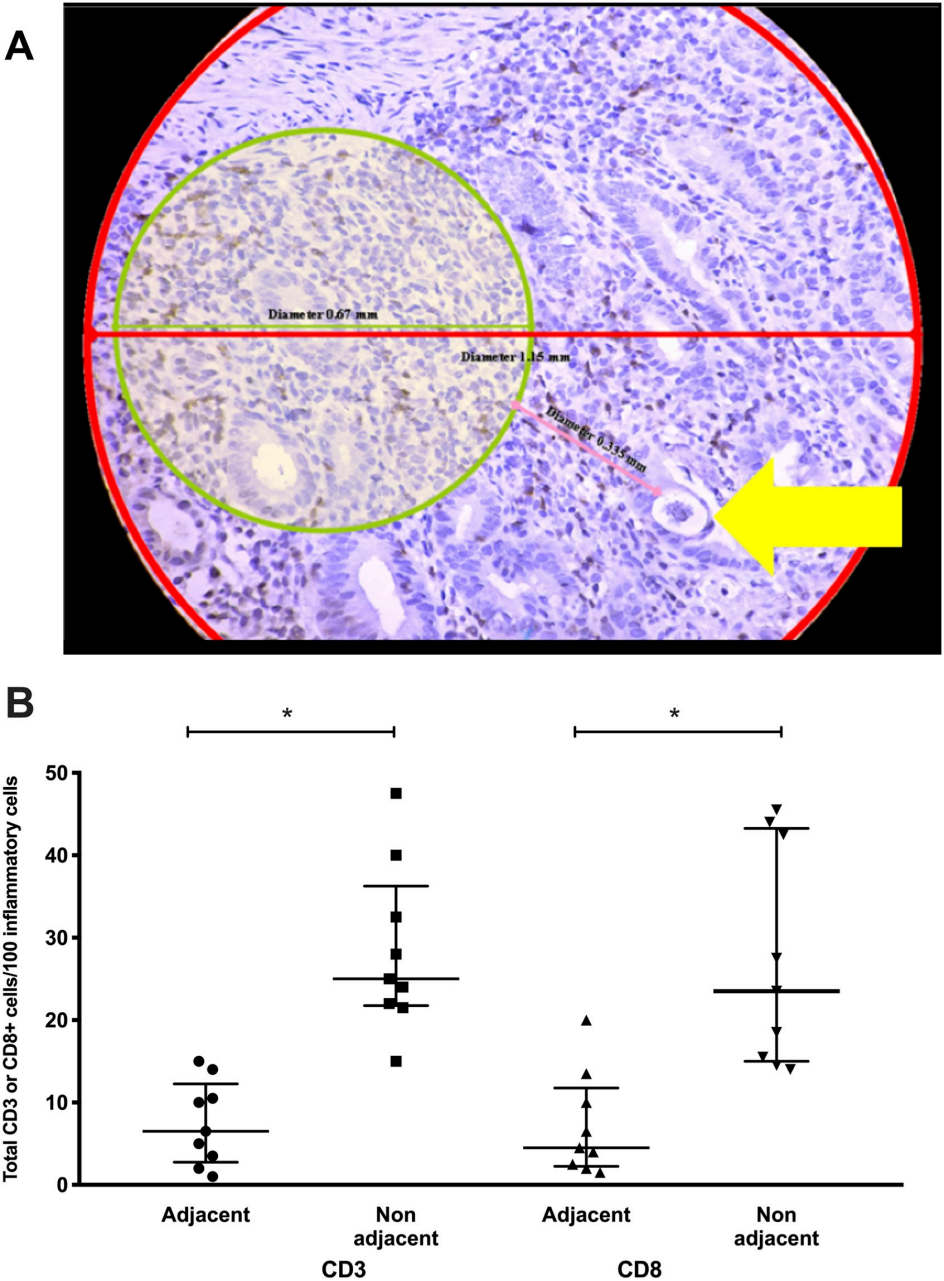
The number of CD3+ T cells and CD8+ cells adjacent and not adjacent to parasites. Panel A. Parasites were identified by microscopy (arrow). Areas within a 0.335 mm radius of the parasite material on the slides were classified as adjacent to the parasite, which was the largest area that allowed characterization of adjacent and non-adjacent cells. Cells in areas adjacent or non-adjacent areas were quantified using ImagePro plus software. Panel B. Slides were stained by immunohistochemistry for CD3+ or CD8+ cells and positive cells were quantified using ImagePro Plus software demonstrating decreased numbers of CD3+ and CD8+ cells in areas adjacent to parasites compared to non-adjacent areas. Long horizontal lines are the medians, vertical line and short horizontal lines demonstrate the interquartile range (*p = 0.03, Mann-Whitney).

**Fig 4:**
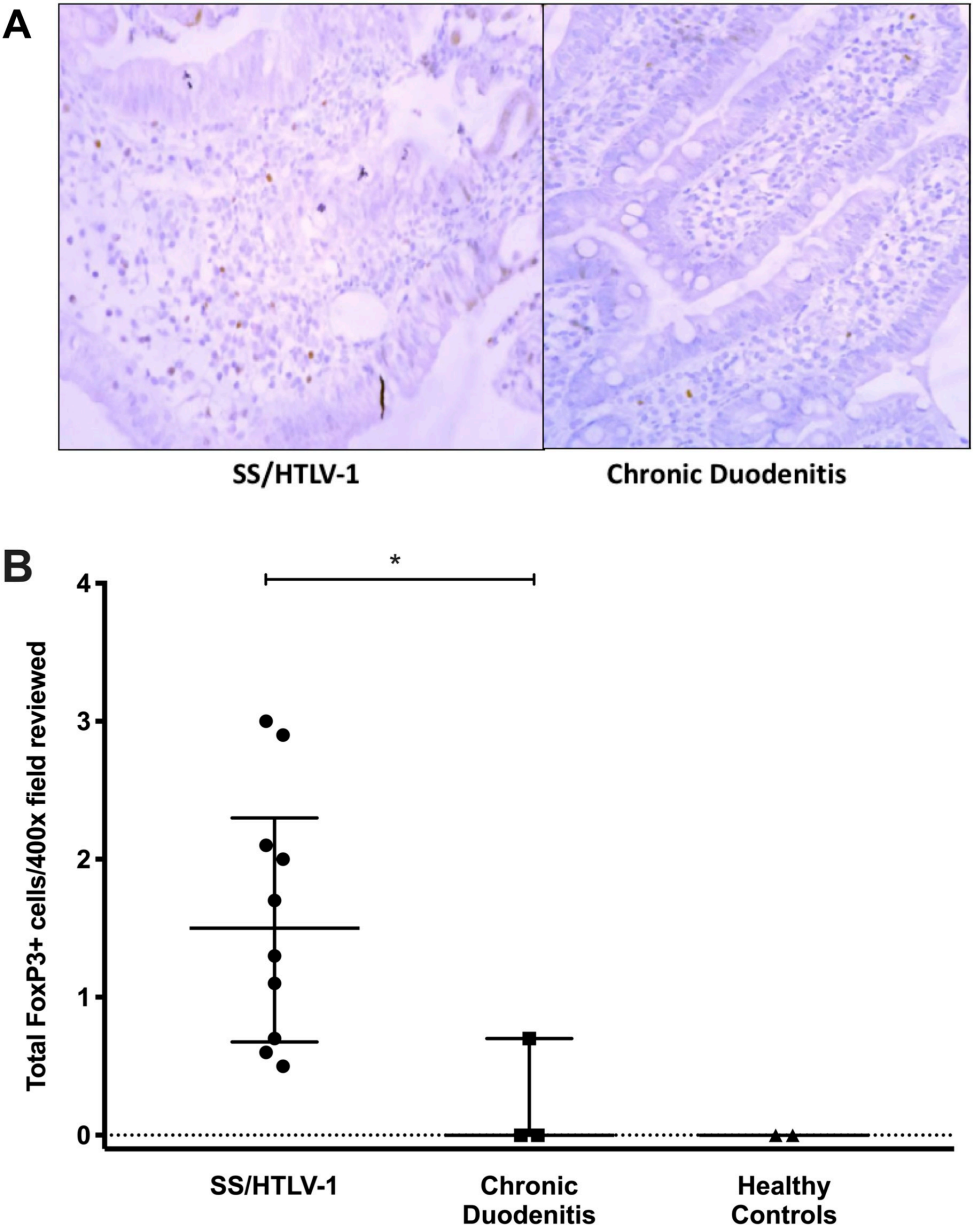
Increased numbers of cells positive for the regulatory T cell marker FoxP3 in HTLV-1 and S*trongyloides* co-infected (SS/HTLV-1) patients. Panel A. Regulatory T cells were identified by immunohistochemistry staining for FoxP3, comparing SS/HTLV-1 patients and controls. Immunohistochemical stains for FoxP3 showing biopsies from an SS/HTLV-1 patient and a control patient (positive cells identified by the brown color). Panel B. FoxP3+ cells were quantitated using ImagePro Plus software. More of the lymphocytes were FoxP3+ in the SS/HTLV-1 patients compared with chronic duodenitis and healthy controls. Long horizontal lines are the medians, vertical line and short horizontal lines demonstrate the interquartile range (*p = 0.03, Mann-Whitney).

In the co-infected subjects, the majority of lamina propria cells were strongly CD3+ (Fig 1B). The number of CD3+ cells from patients with SS/HTLV-1 ranged from 18.3 to 22.4 lymphocytes/100 inflammatory cells (median 20.3) compared to a range of 9 to 12 lymphocytes/100 (median 8.5) in controls with chronic duodenitis (p = 0.04 comparing CD3+ lymphocytes in the lamina propria for co-infected patients compared to chronic duodenitis controls, Fig 1A). We observed a similar total number of CD3+ cells in duodenal biopsies from SS/HTLV-1 compared to chronic duodenitis controls but more than the healthy group (CD3+ cells in the lamina propria SS/HTLV-1: 22.3 [IQR: 19.5–24.2]; chronic duodenitis: 23 [IQR: 13–27.5]; healthy controls: 12.6 [IQR: 11.8–13.3], Mann-Whitney test p = 0.03 comparing lamina propria lymphocytes in SS/HTLV-1 to healthy controls). The median number of CD8+ cells in the duodenal biopsies of SS/HTLV-1 patients was greater than for the two control groups (SS/HTLV-1: 20.3 [IQR: 18.3–22.4]; chronic duodenitis controls: 8.5 [IQR: 9–12]; healthy controls: 12.7 [IQR: 12.5–12.9, Mann-Whitney p = 0.04 comparing SS/HTLV-1 to duodenitis controls, Fig 2A and 2B). For SS/HTLV-1 co-infected patients, fewer of the CD3+ and CD8+ cells were within the villi compared to the crypts (comparing Villi to Crypts in co-infected patients CD3+: Villi: 32 [IQR:29.3–41.3]; Crypts: 11.4 [IQR: 9.8–13.8]; Mann-Whitney p = 0.01; CD8+: Crypts: 26 [IQR:23.5–30]; Villi: 14 [IQR: 11.5–18.3]; Mann-Whitney p<0.003, Fig 1B). There were no differences in distribution of CD3+ and CD8+ cells between the chronic duodenitis and healthy groups (Figs 1 and 2). We measured areas adjacent to the parasites and non-adjacent areas (Fig 3A). For the co-infected patients, there were fewer lymphocytes in areas adjacent to the parasite compared to areas without parasites: CD3+ (adjacent: 6.5 [IQR: 2.8–12.3]; not adjacent: 24.5 [IQR: 20.9–34.4]; Mann-Whitney p = 0.0003 comparing areas adjacent to non-adjacent in co-infected patients, Fig 3B), CD8+ (adjacent: 4.5 [IQR: 2.3–11.8]; non-adjacent: 21 [IQR: 15.3–42.9]; Mann-Whitney p = 0.001 comparing areas adjacent to non-adjacent in co-infected patients, Fig 3B). More of the lymphocytes were FoxP3+ in the SS/HTLV-1 patients compared with chronic duodenitis and healthy controls (Fig 4A, SS/HTLV-1: 1.5 [IQR: 0.7–2.3]; chronic duodenitis: 0 [range 0–0.7]; healthy controls: 0; Mann-Whitney p = 0.03 comparing the proportion of lymphocystes that were FoxP3 positive in co-infected patients to controls, Fig 4B). There were no significant differences between the villi and crypts.

**Fig 5:**
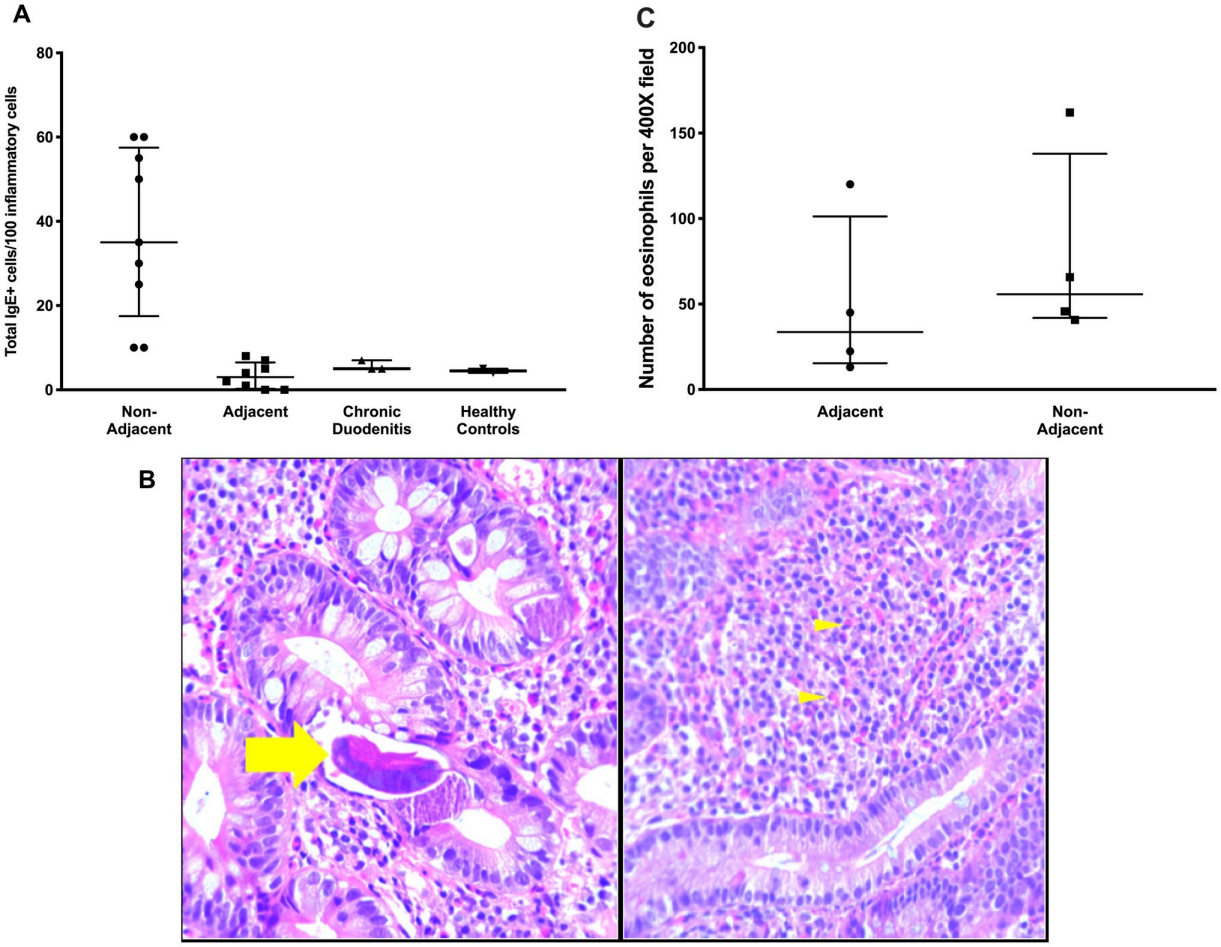
Eosinophils and IgE+ cells in HTLV-1 and S*trongyloides* co-infected (SS/HTLV-1) patients. Panel A. IgE+ cells were identified by immunohistochemistry staining and quantitated using ImagePro Plus software. More of the lymphocytes were IgE+ in the SS/HTLV-1 patients compared with chronic duodenitis and healthy controls (*p = 0.03, Mann-Whitney). Panel B. Three of the SS/HTLV-1 patients had marked infiltration of the duodenum with eosinophils, but there was sparing of sections with parasites. The panels show representative sections containing parasites, but few eosinophils (left, parasite form marked with the arrow) or no parasites and numerous eosinophils (right, some eosinophils marked with arrowheads). Panel C The number of eosinophils per 100x field were counted for fields with or without parasites visible. Eosinophilic infiltrate was less intense in fields with parasites but this was not statistically significant (p = 0.06, Mann-Whitney). Long horizontal lines are the medians, vertical line and short horizontal lines demonstrate the interquartile range.

We observed an increased numbers of IgE+ cells in duodenal biopsies from SS/HTLV-1 patients (Fig 5A). However, there was sparing of areas adjacent to the parasites (adjacent: 3 [IQR: 0.3–6.5]; not adjacent: 35 [IQR: 17.5–57.5]; Mann-Whitney p = 0.001 in biopsies from co-infected patients comparing IgE+ cells in areas adjacent to the parasites to areas not adjacent). In contrast, few cells expressing IgE antibody were observed in the chronic duodenitis and healthy control groups. None of the controls had significant numbers of eosinophils in the biopsies. By contrast, 3 of the SS/HTLV-1 patients had significant eosinophilic infiltration. For those 3, there was a trend towards fewer eosinophils adjacent to the parasites (Fig 5B, adjacent: median 22 [IQR:16–82]; not adjacent 46 [IQR:36–82], Mann Whitney p = 0.06 comparing areas adjacent to areas not adjacent to the parasites in the 3 cases with co-infection and eosinophilic infiltration, Fig 5C).

## Discussion

*Strongyloides* is unique among the nematodes in that it can proliferate within the human host. The host response normally controls the parasite leading to a chronic, smoldering infection. In contrast, patients treated with corticosteroids and those with HTLV-1 co-infection are predisposed to uncontrolled proliferation of the organisms and the hyperinfection syndrome. Granulocytes including eosinophils and neutrophils are thought to play a critical role in controlling the parasite proliferation [10, 15, 16].

HTLV-1 infection is associated with more frequent and severe *Strongyloides* infections [17–20]. In addition, co-infected patients respond poorly to treatments for strongyloidiasis [21]. This is thought to result from a 2 way vicious cycle. For example, *Stronglyoides* co-infection increases HTLV-1 proviral load and expansion of HTLV-1 infected T cells [14, 22]. *Strongyloides* is normally controlled by activation of eosinophils by a T helper 2 immune response [1]. In co-infected patients, HTLV-1 modulates the host immune response blunting the T helper 2 response and IgE production [23, 24]. We previously demonstrated that HTLV-1 leads to increased numbers of Tregs in the peripheral blood of patients presenting with HTLV-1 and *Strongyloides* co-infection [13, 25]. This was associated with a blunted antigen-specific IL-5 response and lower eosinophil counts compared to patients with only chronic strongyloidiasis. In the present study, we studied numbers of lymphocytes and Tregs in the duodenal mucosa of patients with *Strongyloides* and HTLV-1 co-infection.

We noted increased infiltration with T lymphocytes at the site of infection in the duodenum in co-infected patients. However, most of the T cells were CD8+ or expressed the regulatory T cell marker FoxP3+. In murine models, *Strongyloides* infection is associated with the expansion of Tregs, which suppress host expulsion of the parasites [26, 27]. Tregs can blunt Th2 responses including production of IL-5 [13]. Th2 responses to *Strongyloides* are thought to be critical to control the expansion of the parasites. These responses are characterized by the production of IL-5 by Th2 cells, which mediates the expansion and recruitment of eosinophils, and the immunoglobulin switch towards IgE [12]. Increased numbers of eosinophils were found in the duodenal biopsies of SS/HTLV-1 patients. However, fewer eosinophils were noted in areas adjacent to the parasite. This suggests localized inhibition of the eosinophil-mediated response that controls infection. The mechanism of inhibition are unknown. Either Tregs or parasite molecules might inhibit eosinophil recruitment.

IgE is thought to mediate mast cell and eosinophil degranulation, which may lead to parasite expulsion. In addition, antibody may assist eosinophils and other effector cells to kill invasive larvae. Here we demonstrated that IgE expression is increased in the infected tissues, but not in areas adjacent to the parasite. These data are consistent with the suggestion that Tregs may be responsible for blunting local Th2 responses. Similarly, others have noted increased circulating levels IgE and IL-5 in patients with *Strongyloides* only, but not in those co-infected with HTLV-1 [23, 28].

While there were clearly Tregs at the site of infection and decreased number of effector cells (eosinophils, IgE+ lymphocytes) adjacent to the parasites, we were not able to study the function of Tregs directly. In addition, we were not able to identify specimens from patients with just *Strongyloides* or only HTLV-1. Patients with *Strongyloides* only are usually diagnosed by stool studies and do not often undergo endoscopy with biopsy. However, in a study of apparently normal patients with just *Strongyloides* infection, those with mild symptoms had few changes on duodenal histopathology with slight increased infiltration of mononuclear cells [29]. Similarly, patients with HTLV-1 alone do not typically have gastrointestinal symptoms and thus have no indication for endoscopy. Thus, we cannot conclude whether the abnormalities noted were due to immunomodulation from *Strongyloides* per se, from HTLV-1, or from the combination. However, studies of peripheral blood have demonstrated that co-infection amplifies Tregs more than either infection alone [13, 25].

In summary, we demonstrated that patients with *Strongyloides* and HTLV-1 co-infection have increased numbers of regulatory T cells in intestinal biopsies. These cells are preferentially localized to the intestinal crypts. Areas adjacent to the parasites were noted to have decreased numbers T cells, eosinophils, and IgE positive cells, suggesting localized immunosuppression. These data support the hypothesis that regulatory responses in co-infected patients occur within the duodenal mucosa at the site of adult worms and production of larvae and that the regulatory responses blunt host protective mechanisms.
